# *N*-(2-Formamido­eth­yl)formamide

**DOI:** 10.1107/S1600536808038488

**Published:** 2008-11-22

**Authors:** Jin-hui Yang, Yan-xue Chen, Shao-hui Wang, Jian-lei Wang

**Affiliations:** a School of Materials Science and Engineering, Shijiazhuang Railway Institute, Shijiazhuang 050043, People’s Republic of China; b School of Chemical Engineering and Technology, Tianjin University, Tianjin 300072, People’s Republic of China

## Abstract

The complete molecule of the title compound, C_4_H_8_N_2_O_2_, is generated by a crystallographic inversion center. The occurence of N—H⋯O hydrogen bonds results in the formation of a two-dimensional infinite network parallel to the (010) plane. In this plane, the hydrogen bonds define graph-set motif *R*_4_^4^(22) in a centrosymmetric array by the association of four mol­ecules.

## Related literature

For general background, see: Yang *et al.* (2007 ▶). For related structures, see: Goss *et al.* (1996 ▶). For graph-set notation, see: Bernstein *et al.* (1995 ▶); Etter *et al.* (1990 ▶).
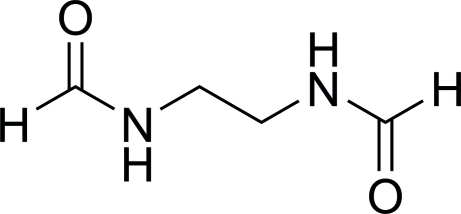


## Experimental

### 

#### Crystal data

C_4_H_8_N_2_O_2_
*M* *_r_* = 116.12
Orthorhombic, 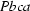

*a* = 8.7138 (17) Å
*b* = 6.6714 (13) Å
*c* = 9.3162 (19) Å
*V* = 541.58 (19) Å^3^
*Z* = 4
Mo *K*α radiation
μ = 0.12 mm^−1^
*T* = 113 (2) K
0.32 × 0.26 × 0.16 mm

#### Data collection

Rigaku Saturn diffractometer
Absorption correction: multi-scan (*CrystalClear*; Rigaku, 2005 ▶) *T*_min_ = 0.964, *T*_max_ = 0.982
2736 measured reflections
467 independent reflections
431 reflections with *I* > 2σ(*I*)
*R*_int_ = 0.035

#### Refinement

*R*[*F* ^2^ > 2σ(*F* ^2^)] = 0.027
*wR*(*F* ^2^) = 0.073
*S* = 1.11
467 reflections
40 parameters
H atoms treated by a mixture of independent and constrained refinement
Δρ_max_ = 0.21 e Å^−3^
Δρ_min_ = −0.15 e Å^−3^

### 

Data collection: *CrystalClear* (Rigaku, 2005 ▶); cell refinement: *CrystalClear*; data reduction: *CrystalClear*; program(s) used to solve structure: *SHELXS97* (Sheldrick, 2008 ▶); program(s) used to refine structure: *SHELXL97* (Sheldrick, 2008 ▶); molecular graphics: *ORTEPIII* (Burnett & Johnson, 1996 ▶), *ORTEP-3 for Windows* (Farrugia, 1997 ▶) and *PLATON* (Spek, 2003 ▶); software used to prepare material for publication: *SHELXL97*.

## Supplementary Material

Crystal structure: contains datablocks global, I. DOI: 10.1107/S1600536808038488/dn2396sup1.cif

Structure factors: contains datablocks I. DOI: 10.1107/S1600536808038488/dn2396Isup2.hkl

Additional supplementary materials:  crystallographic information; 3D view; checkCIF report

## Figures and Tables

**Table 1:** Hydrogen-bond geometry (Å, °)

*D*—H⋯*A*	*D*—H	H⋯*A*	*D*⋯*A*	*D*—H⋯*A*
N1—H1*A*⋯O1^i^	0.844 (15)	2.062 (16)	2.8570 (13)	156.9 (12)

Symmetry code: (i) 
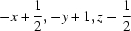
.

**Fig. 1.**
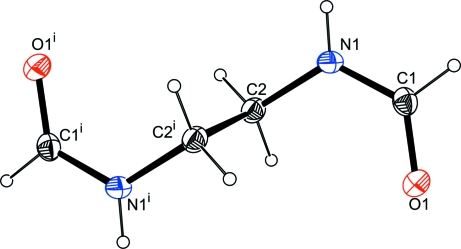
A view of the molecular structure of (I) with the atom-labeling scheme. Displacement ellopsoids are drawn at the 50% probability level and H atoms are shown as small spheres of arbitrary radii. [Symmetry code:(i) 1-x, 1-y, 1-z ]

**Fig. 2.**
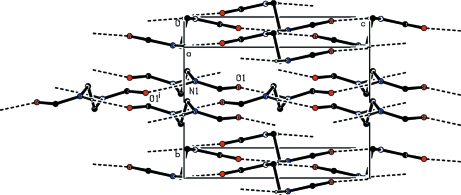
Partial packing view showing the formation of the two dimensional network through N-H···O hydrogen bonds which are shown as dashed lines. H atoms not involved in hydrogen bonding have been omitted for clarity. [Symmetry code: (i) -x+1/2, -y+1, z-1/2]

## supplementary crystallographic information

### Comment

*N*-(2-Formylaminoethyl)formamide is a plasticizer to prepare thermoplastic starch. The mechanical properties of N-(2-Formylaminoethyl)formamide plasticized starch were enhanced compared with the conventional glycerol plasticized one (Yang,*et al.*, 2007).

The molecule of (I) has a center of symmetry at the mid-point of the central C2—C2^i^ bond (Fig. 1).

Intermolecular N—H···O hydrogen bonds link the molecule to form a two dimensionnal network parallel to the (0 1 0) plane. In this plane, the hydrogen bonds define rings by associating 4 molécules displaying graph set motif R_4_^4^(21) (Etter *et al.*, 1990; Bernstein *et al.*, 1995).

Therefore, the OH group of the starch can also form intermolecular O—H···O hydrogen bonds with the *N*-(2-Formylaminoethyl)formamide, the mechanical properties of the plasticized starch is then enhanced.

### Experimental

Methyl formate (500 ml) was placed in a 1000 ml flask cooled by ice-bath and ethylenediamine (250 ml) was slowly added. Subsequently, ice-bath was removed and the mixture was refluxed for 10 h. After standing overnight, the product was isolated by filtration. The solids obtained by filtration were recrystallized from anhydrous ethyl alcohol in 95% yield. Colorless crystals of *N*-(2-Formylaminoethyl)formamide were obtained by slow evaporation of a solution of anhydrous methyl alcohol at 278 k(m.p. 381 k).

### Refinement

The N-bound H atoms were located in a difference map and freely refined with *U*_iso_(H) = 1.2 *U*_eq_(N)], H atoms attached to carbon were positioned geometrically and treated as riding on their parent atoms [C—H distances are 0.93 Å for CH and 0.97 Å for CH_2_ groups, both with *U*_iso_(H) = 1.2 *U*_eq_(C)].

### Crystal data

**Table:**

C_4_H_8_N_2_O_2_	*F*_000_ = 248
*M**_r_* = 116.12	*D*_x_ = 1.424 Mg m^−^^3^
Orthorhombic, *P**b**c**a*	Mo *K*α radiation λ = 0.71073 Å
Hall symbol: -P 2ac 2ab	Cell parameters from 1513 reflections
*a* = 8.7138 (17) Å	θ = 3.1–27.8º
*b* = 6.6714 (13) Å	µ = 0.12 mm^−^^1^
*c* = 9.3162 (19) Å	*T* = 113 (2) K
*V* = 541.58 (19) Å^3^	Block, colorless
*Z* = 4	0.32 × 0.26 × 0.16 mm

### Data collection

**Table:**

Rigaku Saturn diffractometer	467 independent reflections
Radiation source: rotating anode	431 reflections with *I* > 2σ(*I*)
Monochromator: confocal	*R*_int_ = 0.035
*T* = 113(2) K	θ_max_ = 25.0º
ω scans	θ_min_ = 4.4º
Absorption correction: multi-scan(CrystalClear; Rigaku, 2005)	*h* = −10→7
*T*_min_ = 0.964, *T*_max_ = 0.982	*k* = −7→7
2736 measured reflections	*l* = −9→11

### Refinement

**Table:**

Refinement on *F*^2^	Secondary atom site location: difference Fourier map
Least-squares matrix: full	Hydrogen site location: inferred from neighbouring sites
*R*[*F*^2^ > 2σ(*F*^2^)] = 0.027	H atoms treated by a mixture of independent and constrained refinement
*wR*(*F*^2^) = 0.073	*w* = 1/[σ^2^(*F*_o_^2^) + (0.0378*P*)^2^ + 0.1199*P*] where *P* = (*F*_o_^2^ + 2*F*_c_^2^)/3
*S* = 1.11	(Δ/σ)_max_ < 0.001
467 reflections	Δρ_max_ = 0.21 e Å^−^^3^
40 parameters	Δρ_min_ = −0.15 e Å^−^^3^
Primary atom site location: structure-invariant direct methods	Extinction correction: none

### Special details

**Table:**

Geometry. All esds (except the esd in the dihedral angle between two l.s. planes) are estimated using the full covariance matrix. The cell esds are taken into account individually in the estimation of esds in distances, angles and torsion angles; correlations between esds in cell parameters are only used when they are defined by crystal symmetry. An approximate (isotropic) treatment of cell esds is used for estimating esds involving l.s. planes.
Refinement. Refinement of *F*^2^ against ALL reflections. The weighted *R*-factor *wR* and goodness of fit *S* are based on *F*^2^, conventional *R*-factors *R* are based on *F*, with *F* set to zero for negative *F*^2^. The threshold expression of *F*^2^ > σ(*F*^2^) is used only for calculating *R*-factors(gt) *etc*. and is not relevant to the choice of reflections for refinement. *R*-factors based on *F*^2^ are statistically about twice as large as those based on *F*, and *R*- factors based on ALL data will be even larger.

### Fractional atomic coordinates and isotropic or equivalent isotropic displacement parameters (Å^2^)

**Table:**

	*x*	*y*	*z*	*U*_iso_*/*U*_eq_	
C1	0.26367 (12)	0.50564 (15)	0.69236 (11)	0.0165 (3)	
H1	0.1616	0.4744	0.7110	0.020*	
C2	0.45751 (11)	0.59669 (16)	0.51762 (12)	0.0158 (3)	
H2A	0.5102	0.6659	0.5948	0.019*	
H2B	0.4575	0.6835	0.4341	0.019*	
N1	0.30073 (10)	0.55554 (13)	0.56027 (10)	0.0160 (3)	
H1A	0.2325 (15)	0.5444 (19)	0.4965 (17)	0.019*	
O1	0.35499 (8)	0.49743 (11)	0.79307 (8)	0.0209 (3)	

### Atomic displacement parameters (Å^2^)

**Table:**

	*U*^11^	*U*^22^	*U*^33^	*U*^12^	*U*^13^	*U*^23^
C1	0.0155 (5)	0.0165 (6)	0.0175 (6)	0.0003 (4)	0.0024 (5)	−0.0025 (4)
C2	0.0170 (6)	0.0170 (6)	0.0133 (6)	−0.0011 (4)	0.0004 (4)	0.0009 (4)
N1	0.0140 (5)	0.0196 (5)	0.0143 (5)	0.0010 (4)	−0.0027 (3)	−0.0015 (4)
O1	0.0200 (4)	0.0288 (5)	0.0139 (5)	−0.0006 (3)	−0.0002 (3)	0.0012 (3)

### Geometric parameters (Å, °)

**Table:**

C1—O1	1.2314 (13)	C2—C2^i^	1.523 (2)
C1—N1	1.3151 (14)	C2—H2A	0.9700
C1—H1	0.9300	C2—H2B	0.9700
C2—N1	1.4490 (15)	N1—H1A	0.844 (15)
			
O1—C1—N1	124.44 (10)	N1—C2—H2B	109.5
O1—C1—H1	117.8	C2^i^—C2—H2B	109.5
N1—C1—H1	117.8	H2A—C2—H2B	108.0
N1—C2—C2^i^	110.91 (11)	C1—N1—C2	122.41 (9)
N1—C2—H2A	109.5	C1—N1—H1A	117.6 (9)
C2^i^—C2—H2A	109.5	C2—N1—H1A	119.2 (9)

Symmetry codes: (i) −*x*+1, −*y*+1, −*z*+1.

### Hydrogen-bond geometry (Å, °)

**Table:**

*D*—H···*A*	*D*—H	H···*A*	*D*···*A*	*D*—H···*A*
N1—H1A···O1^ii^	0.844 (15)	2.062 (16)	2.8570 (13)	156.9 (12)

Symmetry codes: (ii) −*x*+1/2, −*y*+1, *z*−1/2.
